# Method for Underground Mining Shaft Sensor Data Collection

**DOI:** 10.3390/s24134119

**Published:** 2024-06-25

**Authors:** Artur Adamek, Janusz Będkowski, Paweł Kamiński, Rafał Pasek, Michał Pełka, Jan Zawiślak

**Affiliations:** 1 Faculty of Mining Surveying and Environmental Engineering, AGH University of Science and Technology, 30-059 Kraków, Poland; skala3d@skala3d.pl; 2 Institute of Fundamental Technological Research, Polish Academy of Sciences, 02-106 Warsaw, Poland; 3 KOMAG Institute of Mining Technology, 44-100 Gliwice, Poland; pkaminski@komag.eu; 4 Faculty of Mechanical Engineering and Robotics, AGH University of Science and Technology, 30-059 Kraków, Poland; r.pasek@agh.edu.pl; 5 Robotec.ai sp. z o.o., 00-776 Warsaw, Poland; 6 SKALA 3D, 20-712 Lublin, Poland

**Keywords:** LiDAR, IMU, underground shaft mapping, mine mapping

## Abstract

The motivation behind this research is the lack of an underground mining shaft data set in the literature in the form of open access. For this reason, our data set can be used for many research purposes such as shaft inspection, 3D measurements, simultaneous localization and mapping, artificial intelligence, etc. The data collection method incorporates rotated Velodyne VLP-16, Velodyne Ultra Puck VLP-32c, Livox Tele-15, IMU Xsens MTi-30 and Faro Focus 3D. The ground truth data were acquired with a geodetic survey including 15 ground control points and 6 Faro Focus 3D terrestrial laser scanner stations of a total 273,784,932 of 3D measurement points. This data set provides an end-user case study of realistic applications in mobile mapping technology. The goal of this research was to fill the gap in the underground mining data set domain. The result is the first open-access data set for an underground mining shaft (shaft depth −300 m).

## 1. Introduction

This paper first describes the underground mining shaft LiDAR and IMU data set that was collected for further research purposes. The unique added value of this survey is the ground truth data obtained via an industrial grade geodetic survey in a harsh and, at the same time, extremely dangerous environment. It is not so obvious to repeat such data collection by typical researchers since we should fulfill plenty of safety requirements. Moreover, it is challenging to persuade an underground mining shaft company to share underground mining shaft data due to many legal and security restrictions. For this reason, we claim to provide the first underground mining shaft data set, fulfilling the gap in underground and ground mobile mapping research. We strongly distinguish underground mining shaft data from the underground mining data that are evident in literature. Underground mining data are collected by many researchers [1,2,3,4,5,6,7,8,9], thus it can be considered an already well-investigated area of research. Unfortunately, there is a lack of underground mining shaft data in the literature, since it requires solving the problem of incorporating qualified work at the personnel level. We addressed this issue by using an industrial grade geodetic survey satisfying all safety permissions. For this reason, we claim to provide a novel contribution to the underground mining community. The uniqueness of our data set is

- vertically oriented 3D point cloud (dangerous operation for not qualified personnel),
- highly accurate ground truth obtained with geodetic survey,
- mixed LiDAR data (repetitive scanning pattern, non repetitive scanning pattern).

Shaft mining is a form of underground mining. Shafts are pushed vertically from top to bottom to excavate the ores and minerals. It is also called shaft sinking. It is best suited for concentrated minerals such as iron, coal, etc., which can be found at the depth of the earth’s surface. Shaft sinking refers to shallow shafts and it is different from a deep shaft. The former is sunk for civil engineering projects, and the latter is sunk for mining projects. When the excavation is carried out on the ground surface, it is called a shaft, and if it is underground, it is called a sub-shaft. Inspection of the shaft is crucial for preserving the safety.

**Figure 1:**
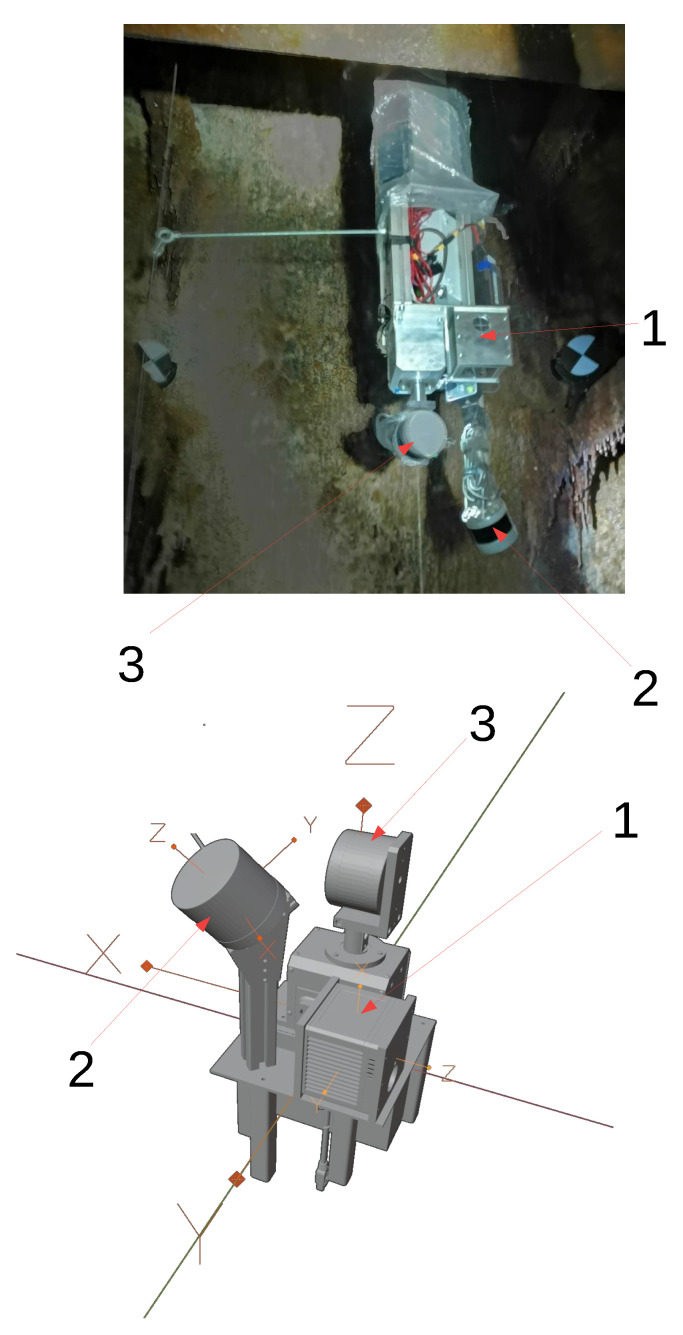
**Top**: the mobile mapping system suspended on the cable over the shaft, **bottom**: Coordinates frames for each sensor. 1—Livox TELE-15, 2—rotated VLP-16, 3—tilted VLP-32c.

**Figure 2:**
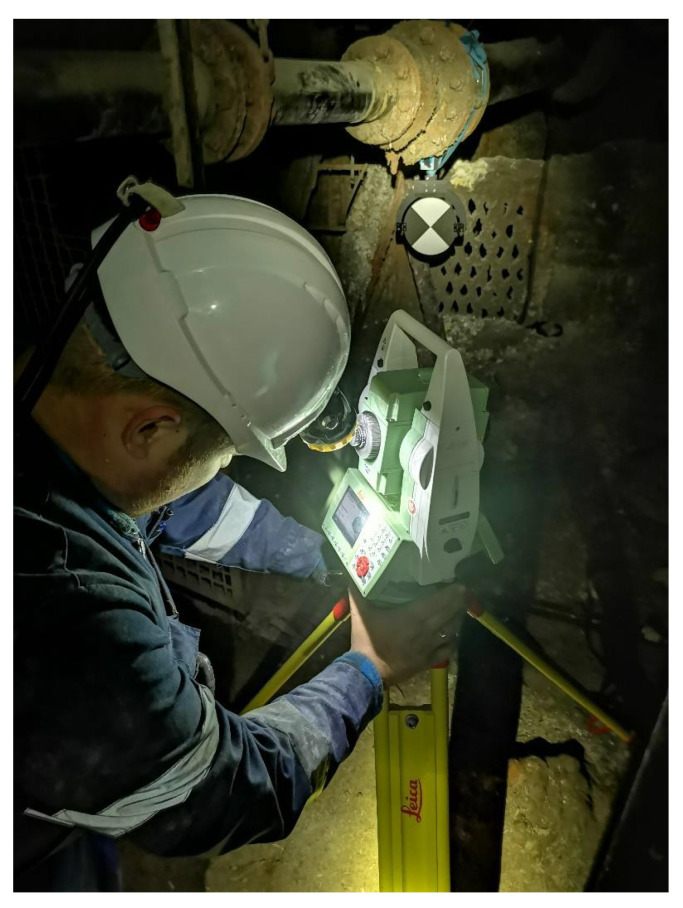
Geodetic measurements with Leica TS11.

**Figure 3:**
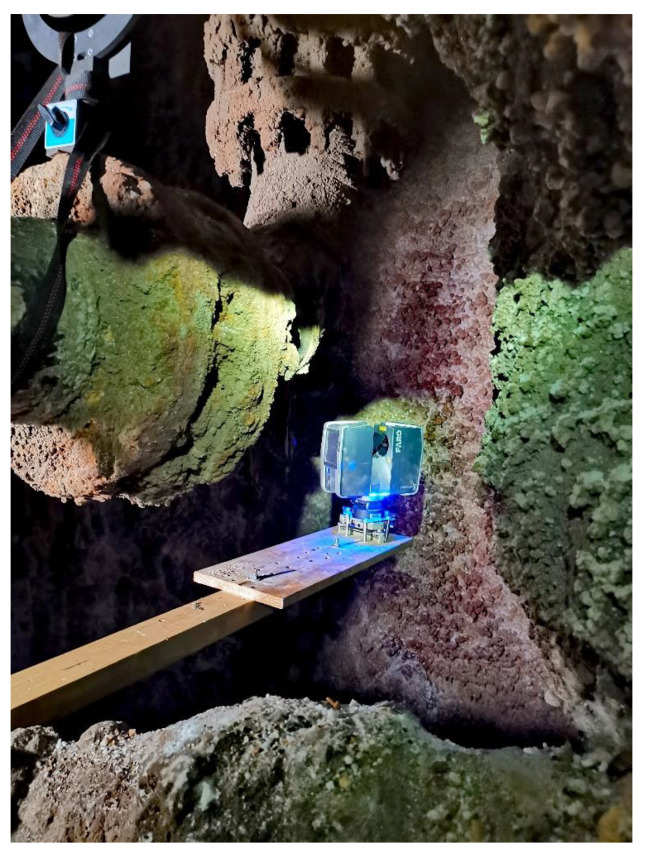
TLS (Terrestrial laser scanning) in the shaft with Faro Focus 3D.

**Figure 4:**
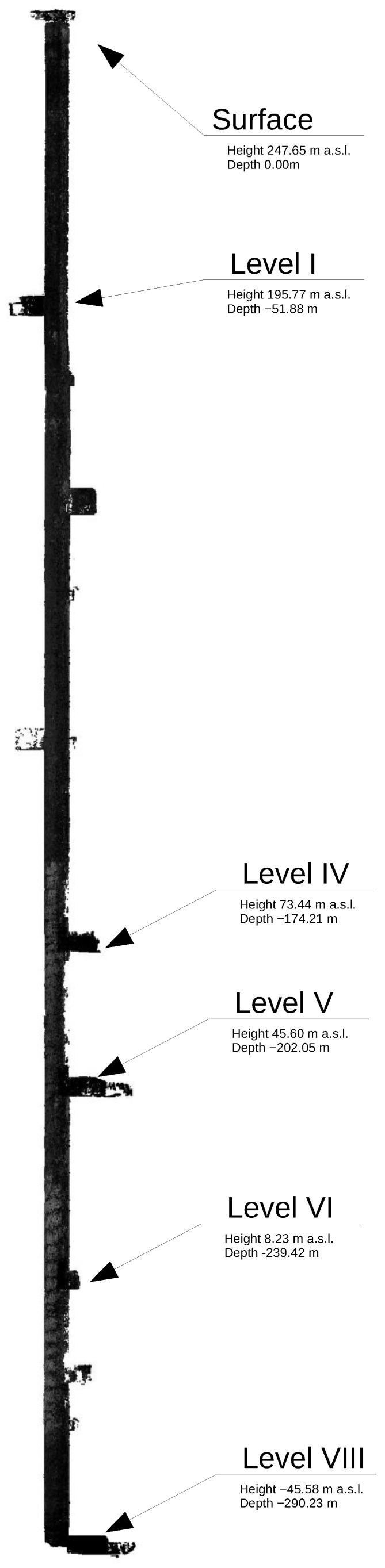
Cross section of the shaft with levels marked.

**Figure 5:**
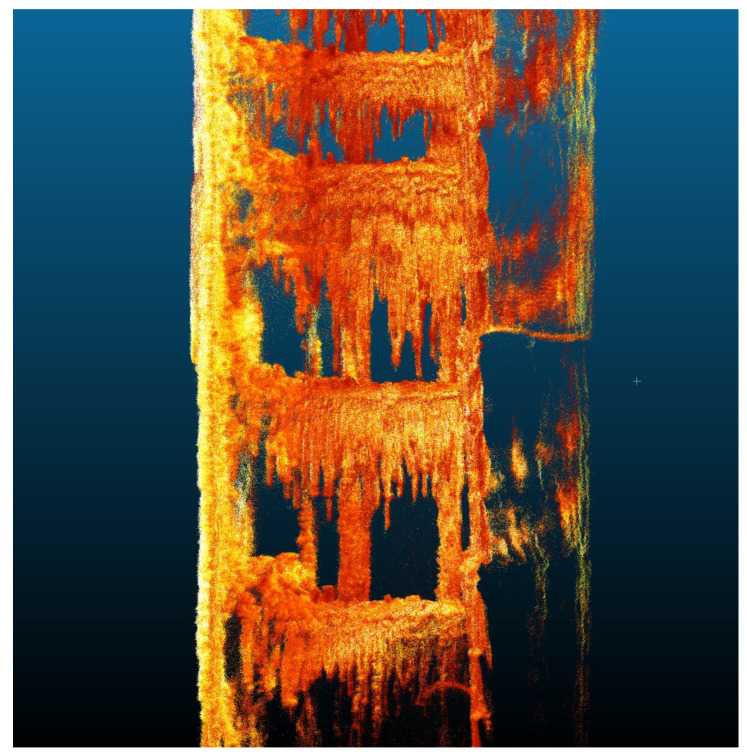
Mobile mapping data of the salt crystal underground mining shaft.

**Figure 6:**
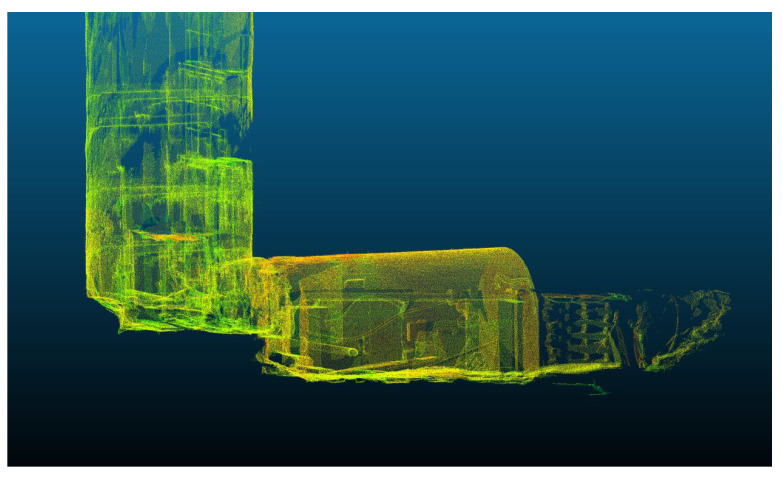
Ground truth terrestrial laser scan from the bottom of the shaft.

Mapping was conducted on November 2021 using the wireline mobile laser scanning platform. The measurements were divided into three parts: classic geodetic measurements aimed at making georeference to the surveys, terrestrial laser scanning and mobile scanning. We collected six stationary scans, as well as the mobile mapping data, by using the mobile mapping system mounted on the metal line that was drained by crane down the underground mining shaft of 300 m depth. The works began with setting out scanning targets at available levels on the shaft structure as reference points. Then, geodetic surveys were obtained to determine the XYZ coordinates of the mounted targets. The position of the targets was determined with an average error from a few to several millimeters, depending on the accuracy of the mine’s network at a given underground level. The uncertainties are given in Table 1. The measurements were made on six levels. The goal of this paper is to share a data set collected with the underground mining shaft mobile mapping system shown in Figure 1. It is equipped with rotated Velodyne VLP-16, Velodyne Ultra Puck VLP-32c, Livox Tele-15, IMU Xsens MTi-30 robotics and geodetic communities. The ground truth data were obtained with a geodetic survey using Leica TS11 (Figure 2) and Faro Focus 3D (Figure 3) over the entire underground mining shaft shown in Figure 4. This data set provides measures obtained in the salt crystal underground mining shaft shown in Figure 5 and Figure 6.

The ground truth data are delivered as 15 ground control points and 6 stationary 3D scans performed on different levels for covering the entire shaft. The purpose of the measurements was to make a detailed and accurate 3D map of the shaft, which is to be renovated and rebuilt. The mining shaft has not been used for years, which makes it impossible to carry out any measurements inside it with the participation of a human. Adamek and Będkowski [10] show that performing classic geodetic surveys using mobile mapping is limited. The basic task for the success of the mapping, in that case, was to perform classical geodetic measurements to create a georeference and calibration base in relation to the existing mine network of geodetic points. Furthermore, a number of stationary scans were made on the surface and on a few underground levels of the mining excavations. The shaft currently has a ventilation function. At the depth of the first 60 m, it has a “barrel” cross-section, and from there down to the bottom, the shaft lining is circular. The shaft has neither built-up guides nor the hoisting machine to slide down. In this situation, it was only possible to perform remote measurements using laser scanning technology to obtain 3D spatial data. Due to technical limitations, it was decided to construct a measuring system that would be lowered into the shaft from the surface. Thus, it provides full coverage of the shaft. All of the data are released and maintained at https://michalpelka.github.io/mine-mapping-dataset/ (accessed on 20 April 2024).

### Motivation

The goal of this research was to fill the gap in the underground mining data set domain. The result is the first open-access data set of an underground mining shaft (shaft depth −300 m). Obtaining such high-quality data in a shaft area is difficult and that it is available for use is rare. There are data sets for underground mining, such as the work by [1], which presents a robotic data set collected from the largest underground copper mine in the world. The sensor measurements were recorded from an approximately two-kilometer traverse of an excavation tunnel. An interesting benchmark by [11] provides underground data acquired with the Riegl VZ-400 terrestrial laser scanner. Another example is a work by [12], providing a data set that encapsulates various complex urban features and addresses the major issues of complex urban areas, such as unreliable and sporadic global positioning system (GPS) data, multi-lane roads, complex building structures and the abundance of highly dynamic objects. Similarly, the data set from [13] relates to the urban scenario, but it contains over 100 repetitions of a consistent route through Oxford, UK, captured over a period of over a year. The main motivation behind our shaft survey, according to our best knowledge, is the lack of such data sets, even though some research in this domain is evidently shown by [14]. For this reason, we claim to fill the gap between available data sets. This work can be used for the study of several aspects in simultaneous localization and mapping [15], LiDAR odometry [16] and mine shaft mapping [10].

## 2. Methods

The procedure of data acquisition is composed of two phases. The first phase was to collect ground truth data with a terrestrial laser scanner and ground control points. The second phase was to use a mobile mapping system to collect mobile data. The mobile mapping data set consists of three LiDARs:

1. Livox TELE-15
2. Velodyne Puck VLP-16 assembled to rotating turntable
3. Velodyne Ultra Puck VLP-32c

And XSens MTi-30 IMU that can be used for motion model generation. The coordinate frames for each sensor are shown in Figure 1 and denoted in Table 2 with respect to Livox TELE-15.

These sensors offer a different set of features. The first sensor has a narrow (16.2 degrees) conical field-of-view that points downwards. It is a design that is meant for automotive use and provides a detection range up to 500 m. The sensor provides its user with non-repetitive scanning. It is not a solid-state design; it consists of an optical system built around rotating Risley prisms. The explanation of this technology is discussed by [17]. The second sensor is low-resolution spinning LiDAR that is enhanced with a turntable. It spins the whole VLP-16 around its horizontal axis. With the calibrated system and synchronized rotation angle, it provides a wide, omnidirectional field of view. The range is 100 m. The last sensor is similar to the second one but has twice as many lasers (channels). It is mounted on the mast and tilted to the side. This setup maintains uniform density with depth. Its field of view is narrow and points to the walls of the shaft. The drawing of the system is shown in Figure 1. The top part of the system contains a computer and batteries. On the very top, the lifting eye bolt is attached, enabling suspension of the system. The whole assembly is carefully lowered using a winch. This geometric configuration of LiDARs enables calibration of the entire system without the need for external patterns since all fields of view are overlapping. Table 3 denotes the basic parameters of the sensor and links to documentation. Table 4 denotes the parameters of the TLS system that was utilized.

## 3. Materials and Data Sources

**Figure 7:**
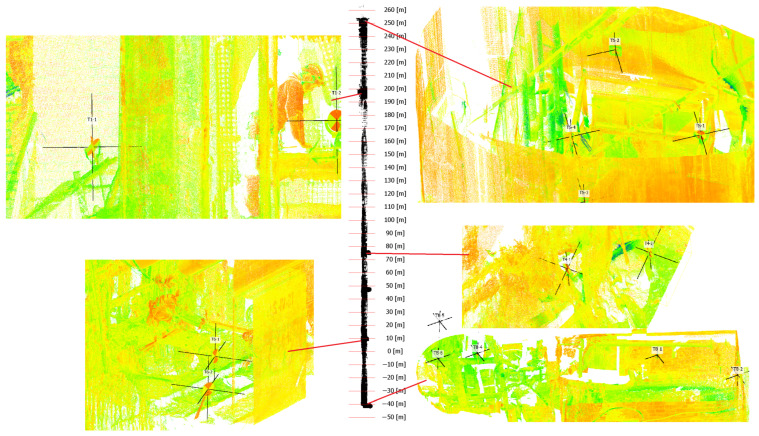
The perspective views of the point clouds with 15 ground control points and 6 Faro Focus 3D terrestrial laser scanner stations. The intersection of orthogonal black lines marks the position of the control point as the center of the target plane commonly used in terrestrial laser mapping. The color map is derived from intensity measurement.

The data set is composed of ground truth and mobile mapping data. The ground truth is composed of 273,784,932 3D measurement points and 15 ground control points, shown in Figure 7. We have chosen the best sensor set looking at the potential cost of the final system. The Faro Focus 3D is a relatively affordable terrestrial laser scanner, providing data with high accuracy. Livox Tele15 is a long range LiDAR (up to 500 m) that can efficiently track the height of the mobile mapping shaft. The rotated Velodyne VLP-16 gives a 360 degree field of view. The Velodyne Ultra Puck VLP-32c provides 2D consecutive profiles of the shaft. IMU Xsens MTi-30 provides acceleration and rotation velocities.

### 3.1. Ground Truth

**Figure 8:**
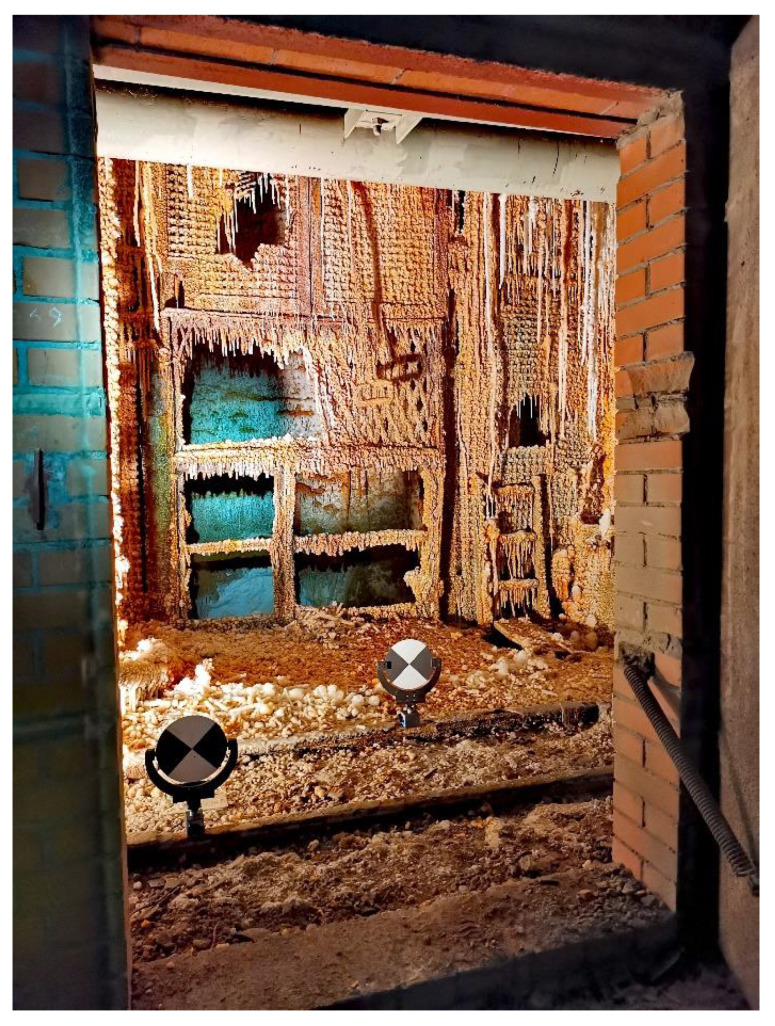
Targets set up at the underground level to perform geodetic surveys and georeference.

**Figure 9:**
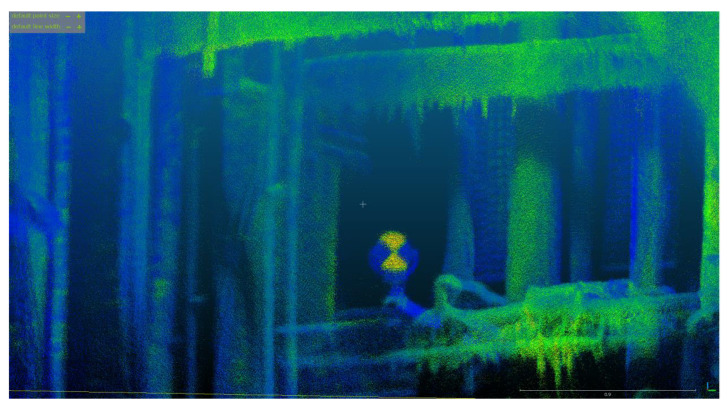
Target seen on the point cloud from the mobile mapping system.

**Figure 10:**
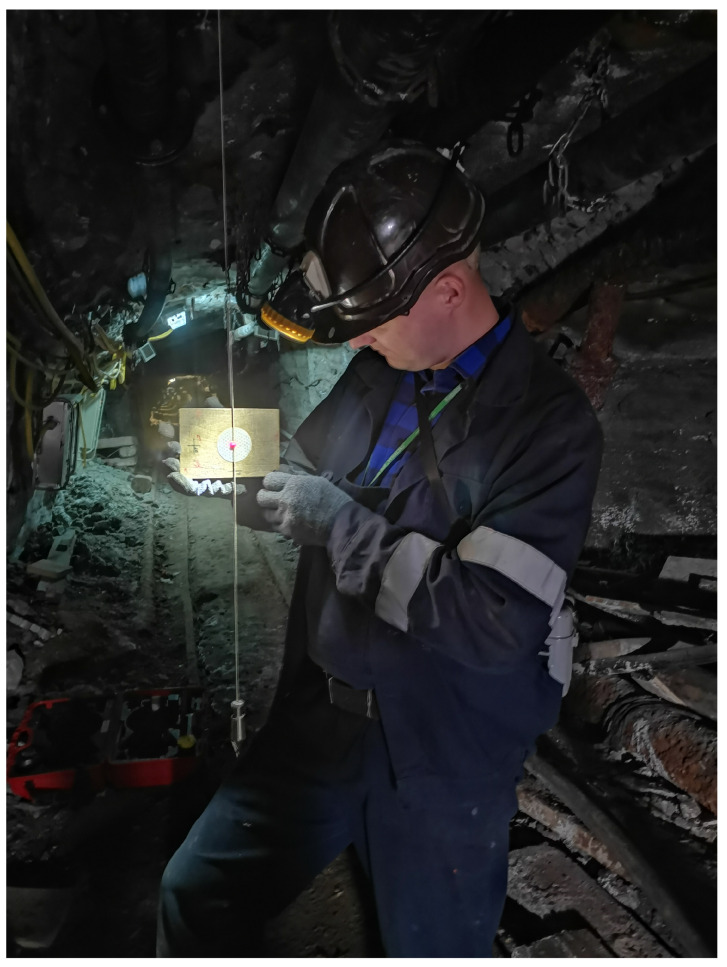
Pointing to the underground control point—showing the factors affecting the accuracy of determining the coordinates of the ground control points.

We provided two types of ground truth data, accurate 3D point cloud data and accurate positions of georeferenced markers obtained with a geodetic survey. The accurate point cloud data are obtained with the state-of-the-art iterative closest point method assuming TLS stations, Livox Tele15 local scans and correspondences between manually picked 3D points and georeferenced markers. We provided the initial guess of the trajectory calculated with the iterative closest point method applied for local point clouds from Livox Tele15. The scanning pattern of the mobile mapping system was to start from the surface and go down to level VIII with a continuous velocity 0.15 m/s. Due to the fact that the shaft was unavailable for direct measurement, as well as it was not possible to perform mobile scanning using the mining survey system [10], a workflow methodology was developed for the purpose of scanning the shaft. The ventilation function of the shaft means that it has to be constantly monitored. Due to this, the geodetic control points still exist within the mine. On the other hand, the age and profile of the mine are determined by the order of accuracy of these benchmarks—up to a few centimeters on the lower underground levels. The measurement methodology assumed the installation of the scanning targets shown in Figure 8 (minimum 2 pcs) in the area of the shaft pipe inlets on each available level, so that their position in relation to the geodetic mine network could be determined. It is also needed to be visible on stationary (Figure 7) and mobile scans (Figure 9) made when the system was exited in the shaft during the mapping. In addition, for the adjustment of the point cloud to the external coordinate system and for a better calibration of the mobile scans, a TLS was planned at the underground levels of mining excavations. The scans were made also in the “light” of the pipe shaft in the “up” and “down” laser scanner head positions. This survey results in ground truth data that are composed of the 15 ground control points shown in Figure 7 with the global coordinates given in Table 1 and the 6 stationary scans given in Table 5. The uncertainty of determining the coordinates of the scanning targets consists of two factors: the accuracy of determining the control points—the geodetic control network (transferring the coordinates from the surface to a given level—this is a measurement made a few or several years earlier) and the accuracy of the measurement of the scanning targets (ground control points) at the time of shaft scanning. Based on our best knowledge, it is difficult to determine with what accuracy the coordinates were laid (put on) underground a few years ago, but we can estimate these values knowing the precision of the measurement techniques used for this purpose. It is assumed that the average error of mechanical plumbing is 2–3 mm per 100 m plus the accuracy of classic geodetic measurements of a single point. Therefore, it can be assumed that the accuracy of the control points should oscillate from 5–6 mm on the first 100 m to 1.5–2 cm on 300 m. Because mistakes add up, to these values, we must add the measurement of the targets mounted during the shaft scanning. In this case, we must take into account the accuracy of determination of the total station position based on the existing control points and the measurement of the centers of the scanning targets. Regardless of the level at which this measurement is made, the average value is 5 mm. The error of pointing to the control point and the accuracy of distance measurements (Figure 10). Taking this into account, we can assume that the uncertainty of the control points ranged from 2–3 mm on the surface up to 2 cm at −290 depth (level VIII). However, this is the optimistic assumption that the underground control point network has been stabilized with adequate accuracy in the past. We believe that this potential issue will be investigated based on this data set in the future.

### 3.2. Data Structure

The data from all the sensors in the mobile system are stored in ROS bag files. Each file contains ten minutes of data streams. During the trial, which lasted for 68 min, the total number of points collected by the system is as follows:

- VLP16: 845 million points.
- VLP32c: 2.30 billion points.
- TELE-15: 970 million points.

The whole data set is divided into multiple rosbag files. Files ‘mine_mapping_001.bag—mine_mapping_007.bag’ contain sensor streams. The file ‘mine_mapping_trajectory.bag’ contains the transformation from ‘map’ to ‘base_link’ with the initial trajectory. The VLP16 and VLP32c LiDARs are configured to spin at a frequency of 10 Hz. The scanning frequency remained constant throughout the trial period. However, the density of points in the output point cloud varies depending on occlusions and the distance from the sensor. The datastreams are organized into the following topics:

- ‘/imu’—Datastream provided by XSens IMU with hardware timestamp.
- ‘/velodyne_rot’—Datastream provided by VLP-16, transformed by rotation, with hardware timestamp.
- ‘/velodyne’—Datastream provided by VLP-32C, in the local coordinate system, with hardware timestamp.
- ‘/livox’—Datastream provided by TELE-15, in the local coordinate system, with hardware timestamp.
- ‘/tf’—Dynamic transformation (rotation) of the VLP16.
- ‘/tf_static’—Static transformation carrying CAD calibration.

The data provided by the LiDARs (VLP-16, VLP-32C, and TELE-15) are organized into messages of type ‘sensor_msgs/PointCloud2’. The hardware timestamp is embedded in every point in the “time” channel. The same hardware timestamps are presented in all headers in the rosbag.

### 3.3. Electronic Design

The important feature of the whole mobile mapping system is its hardware timestamp. The main component here is a microcontroller that provides all devices with hardware timestamps. The implementation is pragmatic—the microcontroller produces a pulse per second (PPS) signal that is fed to all LiDARs. The microcontroller is also responsible for collecting the data from an incremental encoder that was assembled in the turntable (rotated VLP-16 shown in Figure 1 labeled as 3). The hardware synchronization was achieved by following the recommendations from the manufacturers and utilizing the technique presented in the previous work [18]. In this work, we combined LiDAR with a non repetitive scanning pattern with a repetitive scanning pattern. Our contribution is collecting different LiDAR modalities into a common data set.

To summarize, the system incorporates hardware synchronization signals for every measurement device. The microcontroller and LiDARs provide multiple UDP streams that require synchronization, with each LiDAR measurement being timestamped. Additionally, the data from the rotated Velodyne VLP-16 LiDAR need to be associated with the actual rotation angle. To achieve this, a multi-threaded process with multiple open ports collects data from the LiDARs running on the computer. The software development kits (SDKs) (Velodyne Puck VLP-16, Velodyne Ultra Puck VLP-32c; XSpublic from MT Software Suite 2019; TELE-15 Livox-SDK 2.3.0) provided by the manufacturers are utilized to parse the binary data generated by the LiDARs. The implementation leverages associative containers from the C++ standard library, ensuring robustness and efficiency. Finally, the synchronized data stream is serialized and stored on a hard drive using the robot operating system (ROS) framework. The operator of the system has access to telemetric data over a wireless network. Those telemetric data, in the form of web service, allow the operator to start or stop data collection. Remote control was crucial due to safety reasons.

## 4. Results and Analysis

**Figure 11:**
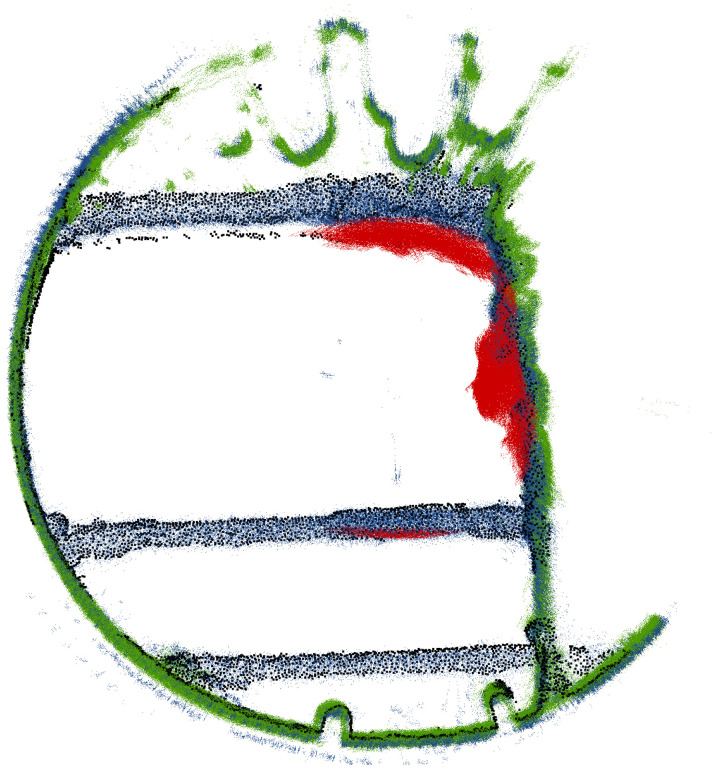
Horizontal cross-section of shaft. Red—Livox TELE-15, Blue—VLP-16, Green—VLP-32c. Black—FARO Focus 3D.

**Figure 12:**
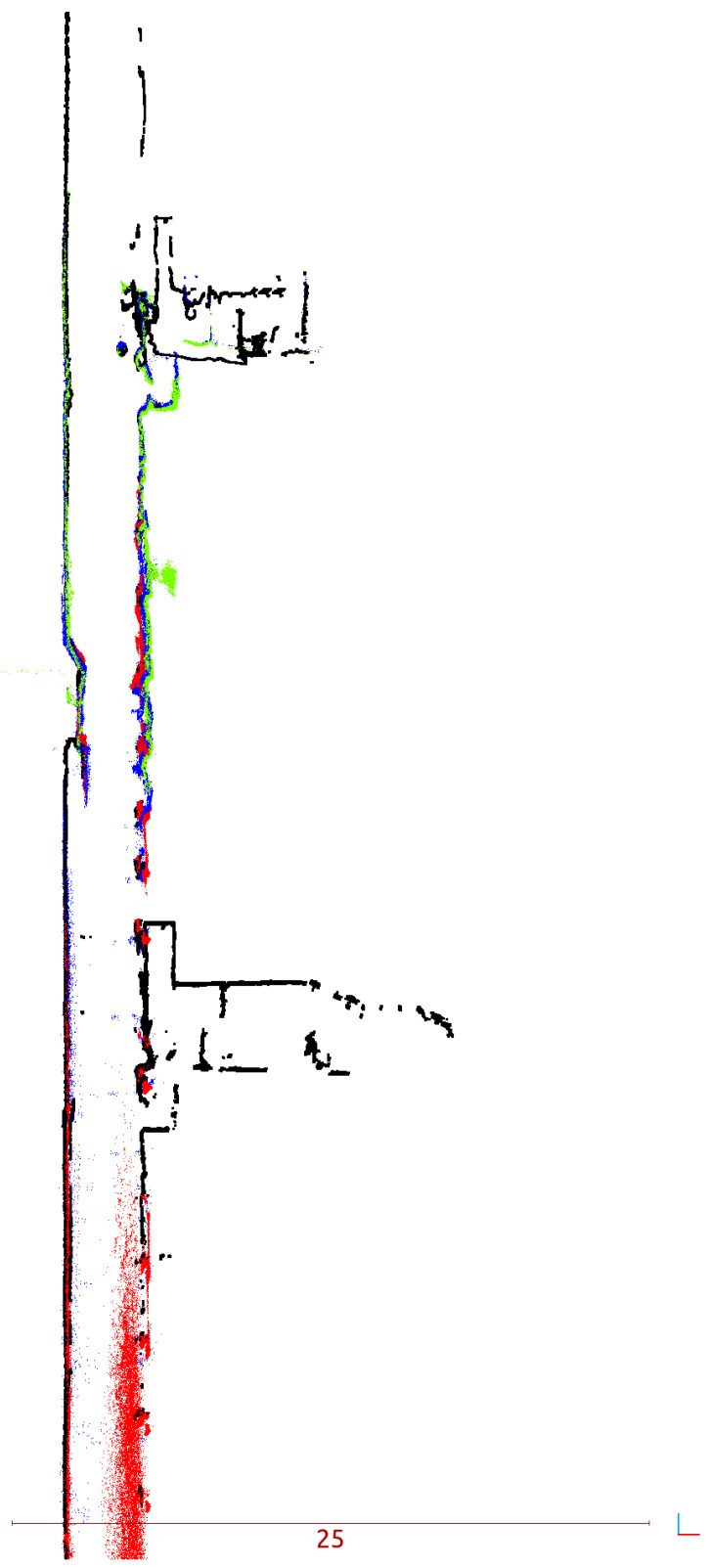
Vertical cross section: color represents the LiDAR in mobile system (Red–Livox TELE-15, Blue—VLP-16, Green—VLP-32c, Black—FARO Focus 3D).

**Figure 13:**
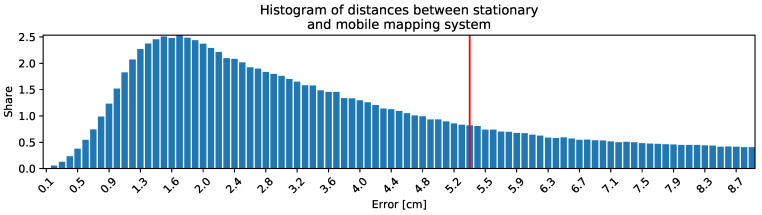
Histogram showing distribution of distances between mobile mapping system (Livox TELE-15, VLP-16 and VLP-32c) and FARO Focus 3D. Marked 90th percentile of distribution.

**Figure 14:**
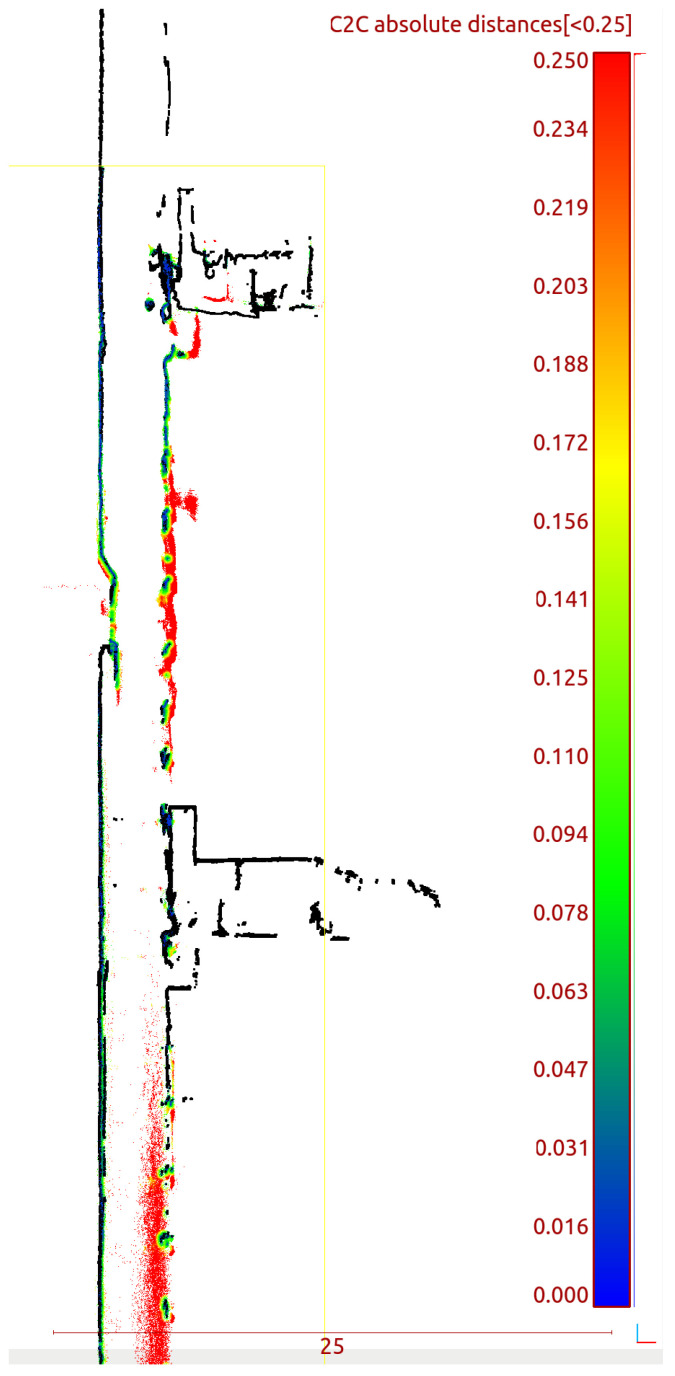
Vertical cross section: distances to ground truth.

This data set is ready for performing benchmarks. Figure 11 shows the horizontal cross-section and Figure 12 shows the vertical cross-section of the shaft with all available LiDAR data. Figure 13 shows the quantitative result: a histogram showing the distribution of distances between the mobile mapping system (Livox TELE-15, VLP-16 and VLP-32c) and ground truth. It can be see that most of the errors are on the centimeter level. Thus, it is a satisfactory result looking from the initial alignment point of view. Researchers can use these results as a starting point for further investigation. Figure 14 shows the vertical cross section with the distance of all mobile mapping data <0.25 m than ground truth. Thus, we claim that this data set is initially aligned to ground truth. The calibration was performed using the ICP algorithm and the used software and data set are available [19].

## 5. Discussion

TLS scanning was performed with the Faro Focus 3D phase scanner and was carried out in such a way as to register the scanning targets and the inner part of the shaft. Similarly to georeferencing, the TLS scanning was performed at the levels: surface, level I, IV, V, VI and the shaft bottom at level VIII. The location of the levels is shown in Figure 4. The scans were performed in the way described above—scanning head at the zenith and then head down at the nadir. In order to obtain a good view of the shaft, the scanner was “ejected” as far as possible into the shaft’s middle on a specially prepared mounting holder (as shown in Figure 3). This concept resulted in greater shaft coverage with panoramic laser data—“precise” scans. The levels selected for TLS scanning are approximately spaced apart at comparable distances, which made it possible to introduce corrections to the determined trajectory of the mobile scanning system. Along with the Z coordinates, the ladder descent inside the shaft—the part accessible for people—from the surface to level I was also scanned, which gave several meters of accurate point cloud inside the shaft. The last stage of measurements was the wireline mobile scanning of the shaft pipe, which was carried out from the surface to the shaft bottom with the use of a winch on a specially designed stand. Additionally, two steel ropes were lowered to guide and stabilize the system passage in the shaft. The mapping was performed twice in two positions of the platform (180 degrees rotation in the second position). Because of the ventilation function of the mine shaft, there was much air flow inside. Therefore, during the mapping, the fan was turned off when the system “passed” through the inlet of the ventilation pipe under the surface to obtain the stability of the platform in the shaft. Nearly 600 GB of laser data were recorded. A video recording of the shaft image with the use of omnidirectional cameras was also made.

## 6. Potential Applications

This work can support many research areas such as surface feature identification [20] in realistic LiDAR scenarios and AI [21]. Our mapping data set provides a LiDAR repetitive scanning pattern and LiDAR non repetitive scanning pattern, thus, various LiDAR Odometry algorithms can be investigated and bench marked due to accurate ground truth. The provided ground truth data can also be investigated for research in accurate terrestrial data registration.

## 7. Conclusions

This paper provides a data set collected with an underground shaft mobile mapping system equipped with rotated Velodyne VLP-16, Velodyne Ultra Puck VLP-32c, Livox Tele-15 and IMU Xsens MTi-30. The ground truth data were acquired with a geodetic survey (15 ground control points and 6 Faro Focus 3D terrestrial laser scanner stations of total 273,784,932 of 3D measurement points). We hope this work improves further mobile mapping system benchmarks without the need for performing such dangerous surveys in hazardous environments such as underground mining shaft where only specialists with certain permissions can work. We believe that our work can improve human safety due to incorporating remotely controlled mobile mapping robots for dangerous tasks.

## Figures and Tables

**Table 1:** The coordinates of ground control points.

Ground Control Point ID	x [m]	y [m]	z [m]	Uncertainty [sigma]
TS-1	−86,345.352	22,671.020	249.098	5 mm
TS-2	−86,346.390	22,672.932	249.391	5 mm
TS-3	−86,347.665	22,669.905	249.503	5 mm
TS-4	−86,347.457	22,671.858	248.701	5 mm
T1-1	−86,347.239	22,668.482	196.697	7 mm
T1-2	−86,347.484	22,671.017	196.918	7 mm
T4-1	−86,346.082	22,672.304	75.892	10 mm
T4-2	−86,345.222	22,673.541	74.974	10 mm
T6-1	−86,345.387	22,673.960	8.564	14 mm
T6-2	−86,344.347	22,674.161	8.564	14 mm
T8-1	−86,345.550	22,678.609	−41.371	20 mm
T8-2	−86,343.015	22,680.587	−41.152	20 mm
T8-4	−86,347.258	22,671.981	−40.004	20 mm
T8-5	−86,347.847	22,670.668	−38.436	20 mm
T8-6	−86,346.088	22,669.629	−38.438	20 mm

**Table 2:** The coordinate frames for each sensor of the mobile mapping system.

Sensor	Type	Extrinsic Calibration	Output
Livox Tele-15	LiDAR	$\left[\begin{array}{cccc} 1 & 0 & 0 & 0\\ 0 & 1 & 0 & 0\\ 0 & 0 & 1 & 0\\ 0 & 0 & 0 & 1 \end{array}\right]$	x, y, z, intensity
VLP-32c	LiDAR	$\left[\begin{array}{cccc} 0.0000 & 0.6428 & 0.7660 & 0.2061\\ 0.7049 & -0.5433 & 0.4559 & 0.0325\\ 0.7093 & 0.5400 & -0.4531 & -0.1567\\ 0.0000 & 0.0000 & 0.0000 & 1.0000 \end{array}\right]$	x, y, z, intensity
VLP-16 rotation base	LiDAR	$\left[\begin{array}{cccc} 0.0 & 0.0 & 1.0 & -0.0678\\ -0.0 & 1.0 & -0.0 & -0.1292\\ -1.0 & -0.0 & 0.0 & -0.0584\\ 0.0 & 0.00 & 0.0 & 1.0000 \end{array}\right]$	x, y, z, intensity
IMU	Inertial Measurement Unit	$\left[\begin{array}{cccc} 0.0 & 0.0 & 1.0 & -0.0680\\ 0.0 & 1.0 & 0.0 & -0.1304\\ -1.0 & 0.0 & -0.0 & -0.0509\\ 0.0 & 0.0 & 0.0 & 1.0 \end{array}\right]$	accelerations, rotation velocities

**Table 3:** Basic parameters of the sensors of the mobile mapping system.

Sensor	Basic Information
Livox TELE-15	Range: up to 500 m
	Range Precision: up to 2 cm
	Laser Wavelength: 905 nm
	Laser Safety: Class 1
	Number of lasers (channels): 1
	Scanning pattern: non repetitive
documentation	https://www.livoxtech.com/tele-15/specs (accessed on 20 April 2024)
Velodyne VLP-16	Range: up to 100 m
	Range Precision: up to 3 cm
	Laser Wavelength: 903 nm
	Laser Safety: Class 1
	Number of lasers (channels): 16
	Scanning pattern: repetitive
documentation	https://velodynelidar.com/products/puck/ (accessed on 20 April 2024)
Velodyne VLP-32c	Range: up to 200 m
	Range Precision: up to 3 cm
	Laser Wavelength: 903 nm
	Laser Safety: Class 1
	Number of lasers (channels): 32
	Scanning pattern: repetitive
documentation	https://velodynelidar.com/products/ultra-puck/ (accessed on 20 April 2024)
Xsens MTi-30	Angular resolution 0.05 deg
	Repeatability: 0.2 deg
	Static accuracy(roll/pitch): 0.5 deg
	Static accuracy(heading): 1 deg
	Dynamic accuracy: 2 deg RMS
documentation	https://shop-us.xsens.com/shop/mti-10-series/mti-30-ahrs/ (accessed on 20 April 2024)

**Table 4:** Basic parameters of utilized TLS.

Sensor	Basic Information
FARO Focus 3D	Range on white surface:
	up to 150 m
	Range on black surface:
	up to 50 m
	Range precision on white surface:
	up to 0.1 mm
	Range precision on black surface:
	up to 0.7 mm
	Angular accuracy: 19 arcsec
	Accuracy of 3D point at
	10 m: 2 mm
	Accuracy of 3D point at
	25 m: 3.5 mm
	Laser Wavelength: 1553.5 nm
	Laser Safety: Class 1
documentation	https://www.faro.com/en/Resource-Library/Brochure/FARO-Focus-Premium (accessed on 20 April 2024)

**Table 5:** Number of measurement points for each stationary scan and elevation ranges.

Stationary Scan	Number of 3D Points	Elevation min [m]	Elevation max [m]
Surface	48,494,798	192.57	254.2
Level 1	48,116,790	178.72	254.18
Level 4	24,305,044	38.66	112.31
Level 5	40,505,015	−4.62	112.28
Level 6	63,493,077	−41.26	58.65
Level 8	48,870,208	−43.37	−7.39

## Data Availability

The project is released and maintained at https://michalpelka.github.io/mine-mapping-dataset/ (accessed on 20 April 2024).

## Abbreviations

The following abbreviations are used in this manuscript:

AI	Artificial intelligence
GPS	Global positioning system
TLS	Terrestrial laser scanner
IMU	Inertial measurement unit
LiDAR	Light detection and ranging
PPS	Pulse per second
ROS	Robot operating system
SDK	Software development kit
UDP	User datagram protocol
